# Supplementary material for: Haploidy in somatic cells is induced by mature oocytes in mice
Source: Commun Biol. 2022 Jan 25;5:95. doi: 10.1038/s42003-022-03040-5 (PMC8789866; doi:10.1038/s42003-022-03040-5)
Supplement: Supplementary file 2 — Supplementary information [file 42003_2022_3040_MOESM2_ESM.pdf]

a

Type of PN & PPB	Frequency (N)	Possible explanation
2PN/1PPB	32% (35)	Somatic chromosomes were segregated and extracted.
2PN/0PPB	41% (44)	Somatic chromosomes were not segregated and not extracted, or pseudo polar bodies (PPBs) were disappeared after extraction.
1PN/1PPB	11% (12)	Somatic chromosomes were not segregated and extracted.
3PN/0PPB	5% (5)	Somatic chromosomes were segregated and not extracted.
1PN/0PPB	11% (12)	Somatic cell was not fused with cytoplast. Only sperm PN was formatted.

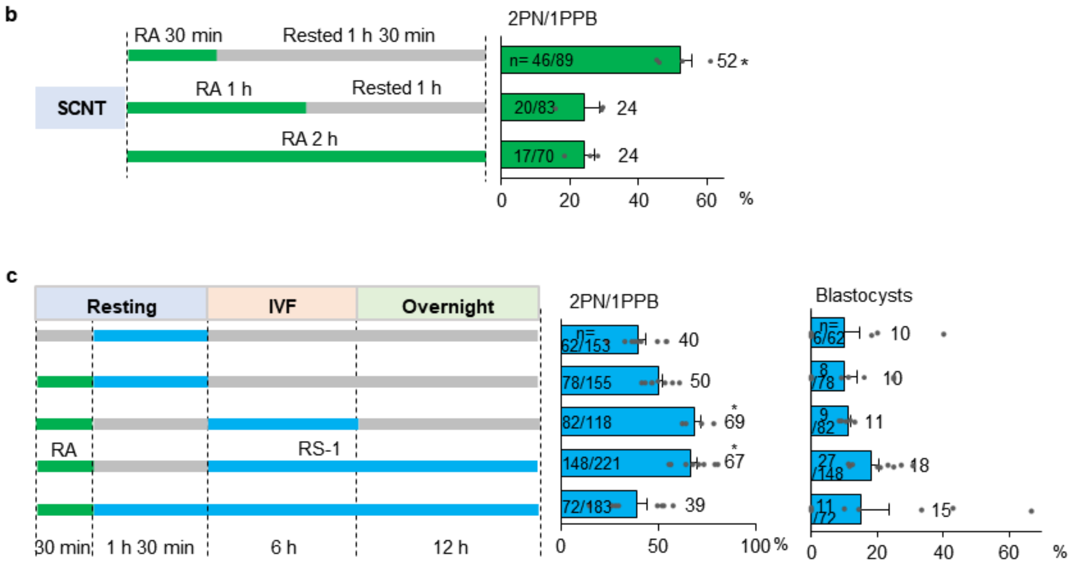

Supplementary Fig. 1. Optimization of the somatic cell haploidization protocol. a, Frequency of various types of SH-zygotes with fasudil treatment. 73% SH-zygotes were formed 2PN and only 32% showed 2PN/1PPB. **b,** Optimization of RA treatment. SCNT oocytes were rested for 2 h before IVF supplemented with RA for 30 min, 1 h, or 2 h. The 30 min-treated groups showed significantly increased 2PN/1PPB formation ($P < 0.05$, by ANOVA with Tukey analysis). **c,** Optimization of RS-1 treatment. RS-1 was treated during the resting time, IVF, and overnight after IVF. Treatment during IVF and overnight resulted in a significantly higher rate of 2PN/1PPB ($P <$ 0.05, by ANOVA with Tukey analysis). For panels b and c, 'n' in 2PN/1PPB and blastocysts graphs represents the number of 2PN/1PPB embryos/the number of fertilized embryos and the number of blastocysts/the number of 2PN/1PPB embryos, respectively. 3-4 technical replications

for panel b for each group. 5-9 technical replications for panel c for each group. mean ± s.e.m. *, $p < 0.05$.

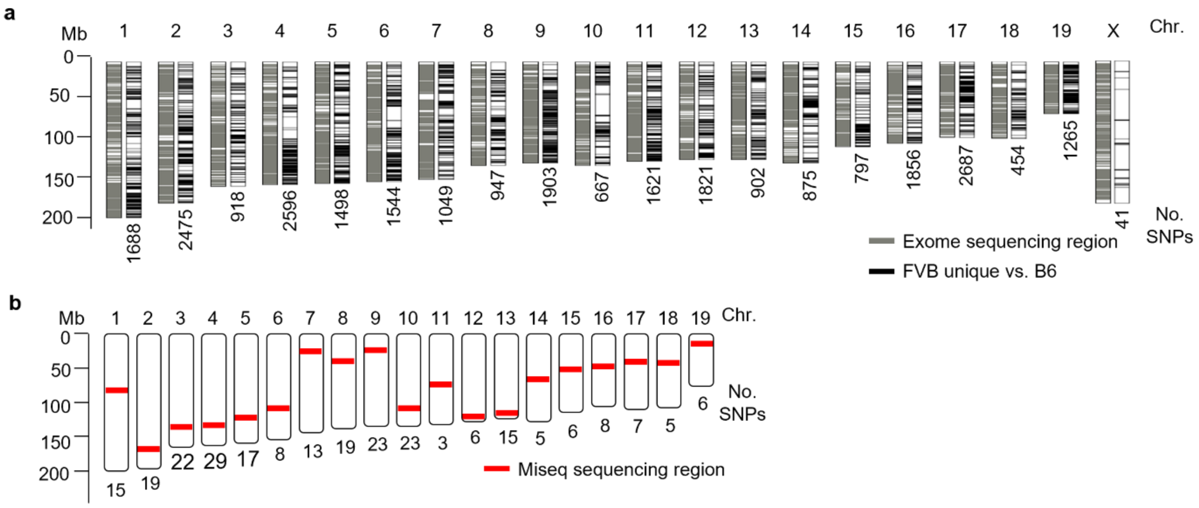

c **The primer sequences used for Miseq analysis.**

Chr.	F primer	R primer	Size (bp)
1	GGAAGGCACCTTAACTCTCAAG	CACCACACGTGTAACTAGCAAG	1,389 bp
2	GGCAGTGTAGCCTAGTGATG	GTTCCCTTCCCAACAGAGAT	1,200 bp
3	GACCTTCCAGGTTCCGGTTACGT	CGTGACTTCACTGTTCTCCTGA	1,439 bp
4	CCATCCTGTGCCTGTTTATGAGTG	GAGACCCATCAGTGGTCTTGAGTG	1,522 bp
5	GCAGGAGCCACAAATAAACGTG	GGAGTCTCACAGGATGTGACTTT	1,333 bp
6	GTAAAGTTGCCAGGGGAGCTGCAA	GACACACAGGTGGAAGCAATAC	1,307 bp
7	GGAGGATGATAAGGCATAGTCGCT	CAGGTTCGAAGCACAGTGAGTGCA	1,479 bp
8	CTCCAAACACTCACCGACTGT	GGACTTATGGTCCACTCTATGC	1,420 bp
9	CGCTGCTGTGATCAGGATTAGA	GTCCAGGAGTGGTTTAGTGGTA	1,437 bp
10	GGGTCTTTGGATTGTAGCACGTTT	GCTTACTCTTGCTACCCATAGCTT	1,426 bp
11	GGAGCTTCTCTTCTCAGGAACCT	GGCTGGCTTCCTGATGCAAGTT	1,439 bp
12	GCACTGGTAGAGTATGTCCTTGT	CTGCTGCTTTGTGATCGAGCAT	1,344 bp
13	CCCGTGAGCAAGTACATCTTAGAG	GCATCCTCCACGATGACAATGGA	1,412 bp
14	GAAAGAGCCCTTGCCTTCTTGC	GTCAGCCAGGTTACATAGCAAG	1,439 bp
15	GAAGCAGCCTTCCCTGGAGTCTCA	GGAGATGCATCTCGGAATAGTGGA	1,119 bp
16	CCACATCCCGGGTAGTGACAAT	GGCGAGGAATGCCCAAAGAGAA	1,362 bp
17	GGACTCAGGAGATTCCCAGTTC	GTGTCAGAGACAGTGTTTCTCCC	1,246 bp
18	GGGGAGAACCGAGCATCAAAGA	GAGCTCAGTACTCTTGCTGGAA	1,344 bp
19	GGACTCCAGTAGAACCTTCACA	CAGCTCTGTTGTAGCTCCCATA	1,410 bp

Supplementary Fig. 2. SNPs analysis for detection of chromosome segregation by WES and Miseq. a, Chromosome maps of the exome sequencing region and FVB-specific SNPs (single-nucleotide polymorphisms) in each chromosome. The FVB-specific SNPs were against that of C57BL/6 (B6). The centromeres were located on top of each chromosome. The numbers below the chromosomes indicate the number of FVB SNPs of each chromosome. **b,** Regions of the sequencing area by Miseq. Red bars indicate the Miseq sequencing region of 1,100~1,500 bp in

each chromosome. SNP values show the number of FVB SNPs in the sequenced area of each chromosome. The numbers below the chromosomes indicate the number of FVB SNPs in the sequencing area. **c,** The primer sequences used for Miseq analysis.

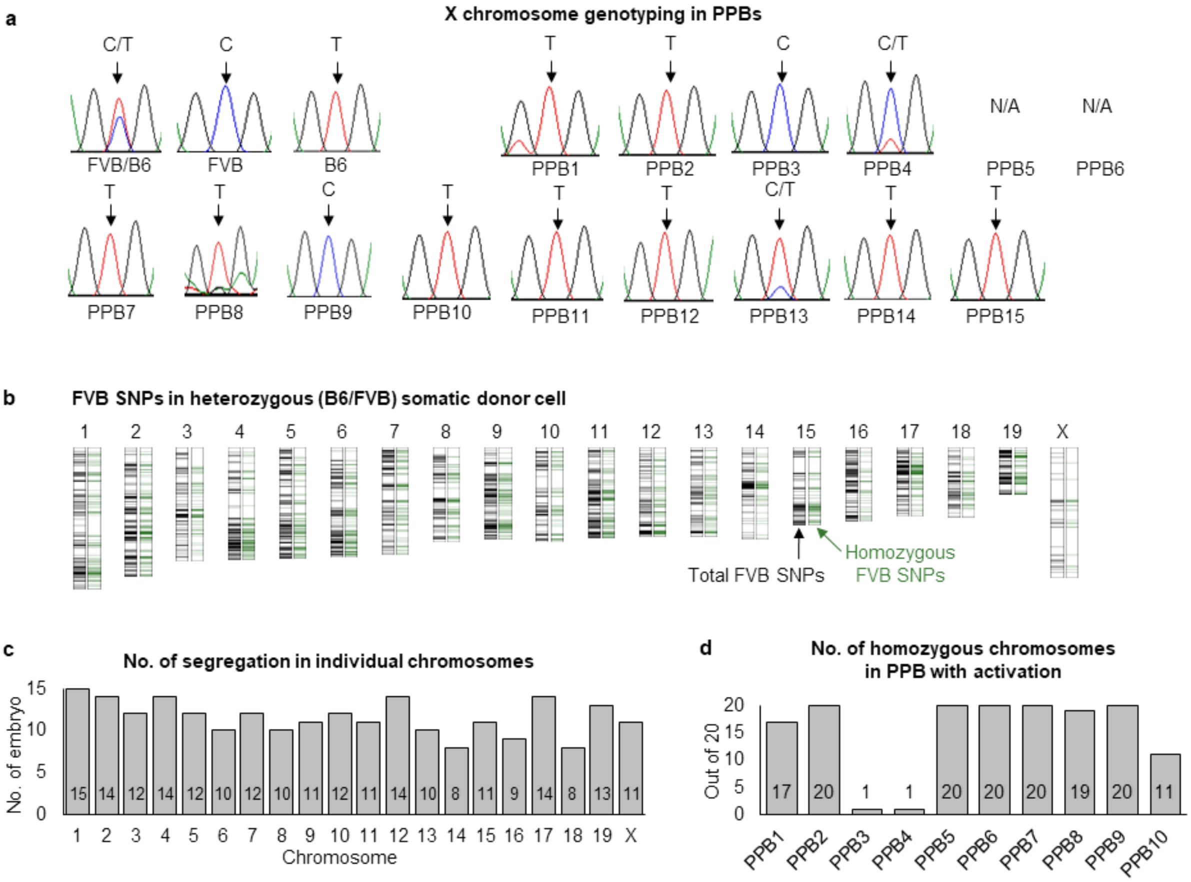

Supplementary Fig. 3. Chromosome segregation pattern in SH embryo. a, X chromosome genotyping in PPB by Sanger sequencing. PPBs showed homozygous (FVB or B6) or heterozygous peaks in chromatography. N/A means the absence of PCR amplicons. **b**, FVB SNPs detected in heterozygous (B6/FVB) somatic donor cell. Black or green bars indicate total FVB or homozygous SNPs, respectively. **c,** The frequency of segregation of individual chromosomes. Chromosome 1 was segregated in all 15 embryos, whereas the other 19 chromosomes were separated into 8 to14 embryos. **d,** The number of homozygous chromosomes in the PPB of chemically activated SCNT oocytes using FVB/B6 donor cells. Among 10 PPBs, five PPBs were detected to be of FVB or B6 homozygous genotype in all chromosomes.

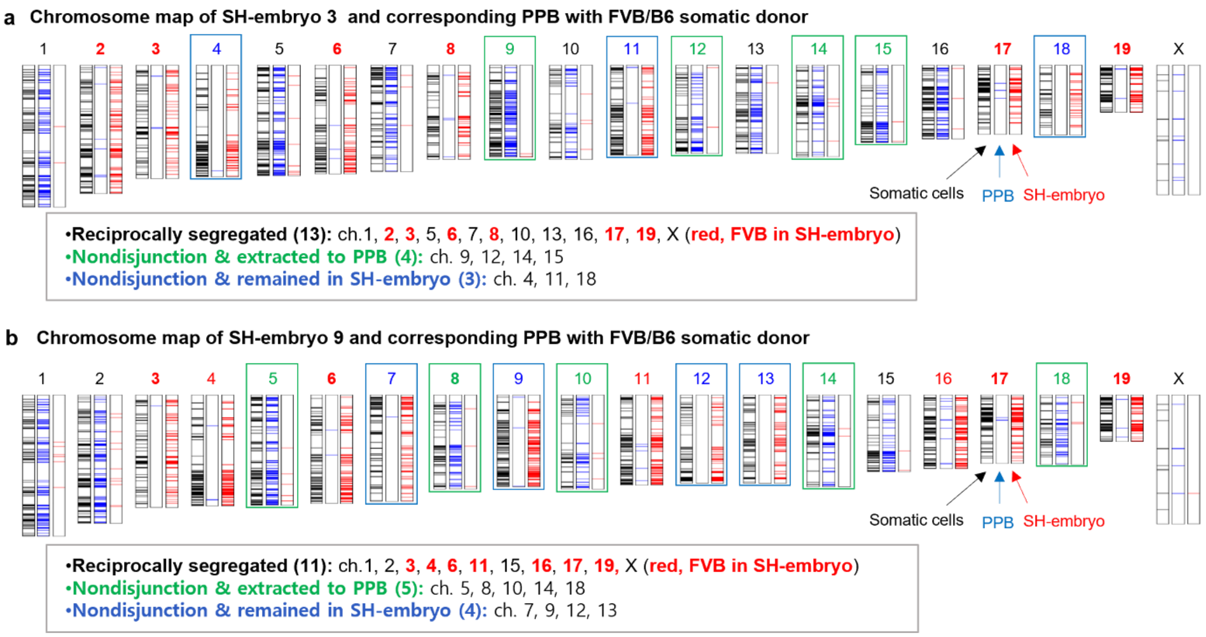

Supplementary Fig. 4. Somatic chromosome segregation to SH-embryos and corresponding PPBs with FVB/B6 somatic donor. In SH-embryo 3 (a) and 9 (b) and their corresponding PPB, 13 and 11 chromosomes were segregated reciprocally. The remaining chromosomes were non-disjunct and extracted to PPB (green boxes) or remained in SH-embryos (blue boxes). 6 (in SH-embryo 3) and 7 (in SH-embryo 9) chromosomes contained the FVB genome (red number above the chromosomes). Black bars indicate FVB SNPs detected in FVB/B6 somatic cells. SNPs on chromosomes with few SNPs could be a sequencing error.

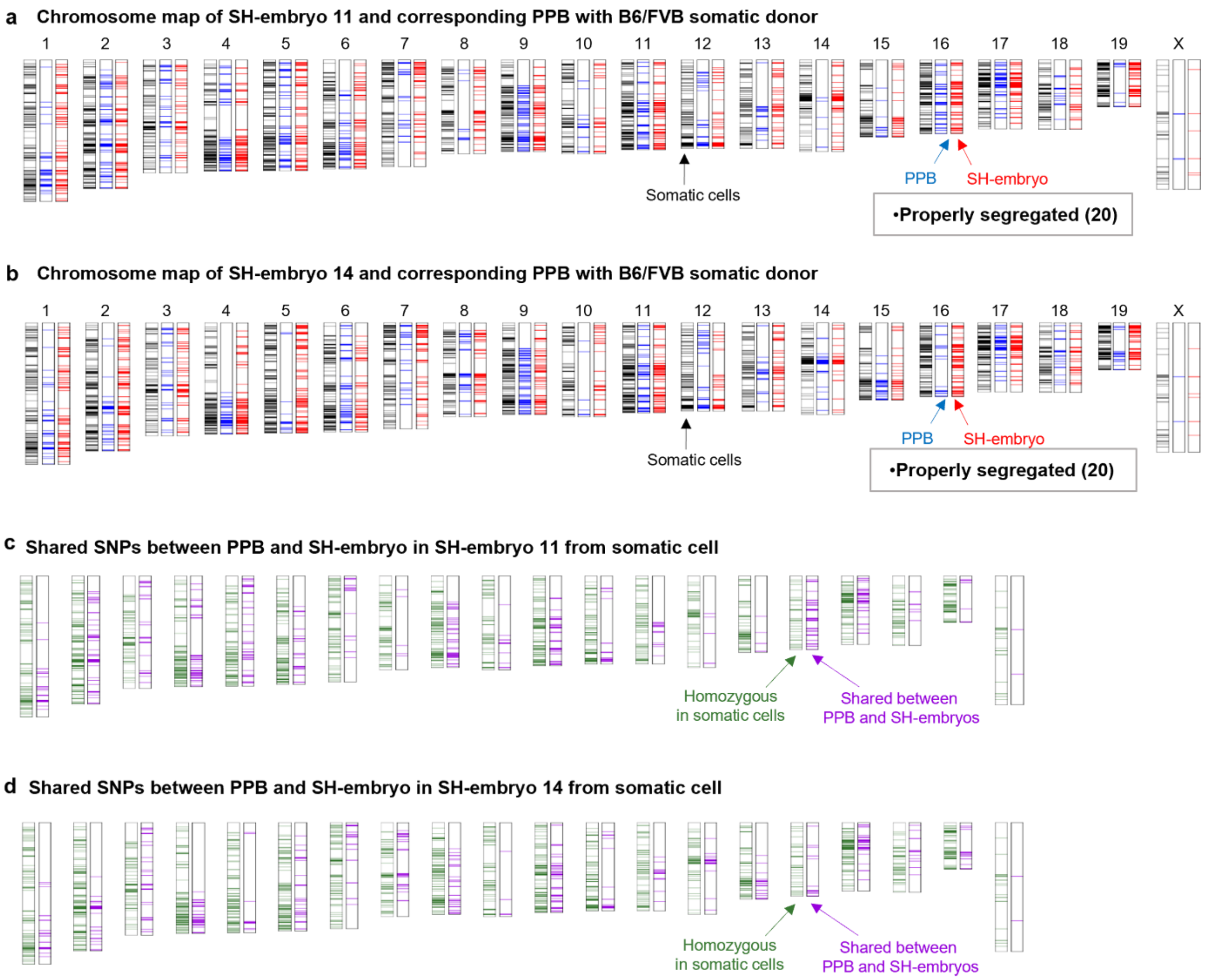

Supplementary Fig. 5. Somatic chromosome segregation to SH-embryos and corresponding PPBs with B6/FVB somatic donor. a and b, Chromosome map of SH-embryo 11 and 14 and their corresponding PPB with B6/FVB somatic donor. Black bars indicate FVB SNPs detected in B6/FVB somatic cells. **c and d,** Shared FVB SNPs between PPB and SH-embryo from a somatic cell in SH-embryo 11 and 14. The green bars indicate homozygous SNPs in somatic donor cells. Purple bars mean shared FVB SNPs between PPBs and corresponding embryos. Analysis of PPBs and embryos could have limitations such as WGA or technical errors.

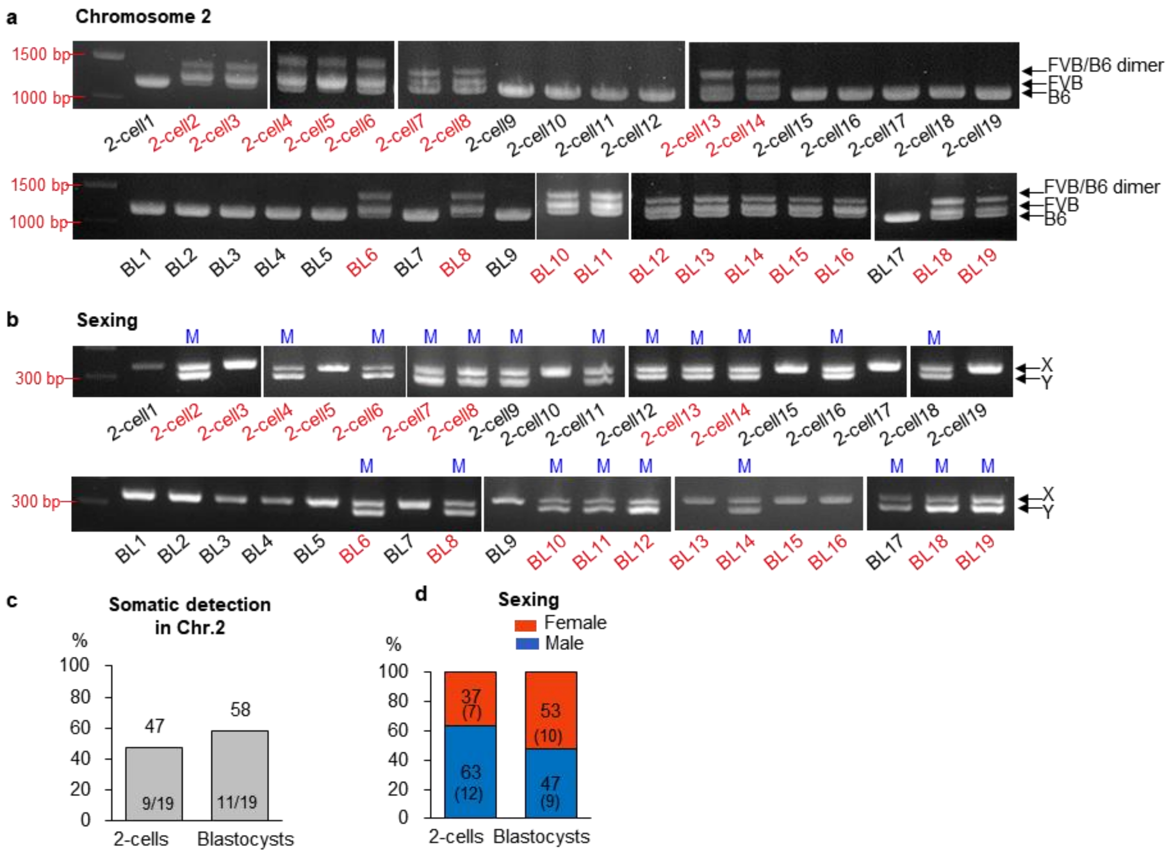

Supplementary Fig. 6. Screening of somatic origin and sex identification in SH 2-cells and SH-blastocysts. a, Screening of chromosome 2 to detect somatic origin. Nine 2-cell embryos and eleven blastocysts (red) were detected with the FVB (somatic origin) genome among nineteen 2-cells and nineteen blastocysts, respectively. **b,** Sex identification by PCR. Letter M above the gel image means male. **c,** The frequency of somatic detection in chromosome 2 of SH 2-cells and SH-blastocysts. Nine 2-cells (47%, 9/19) and eleven blastocysts (55%, 11/19) harbored the FVB genome in chromosome 2. **d,** The sex ratio of the SH embryos. 63% (12/19) and 47% (9/19) of SH were male embryos in SH 2-cells and SH-blastocysts, respectively.

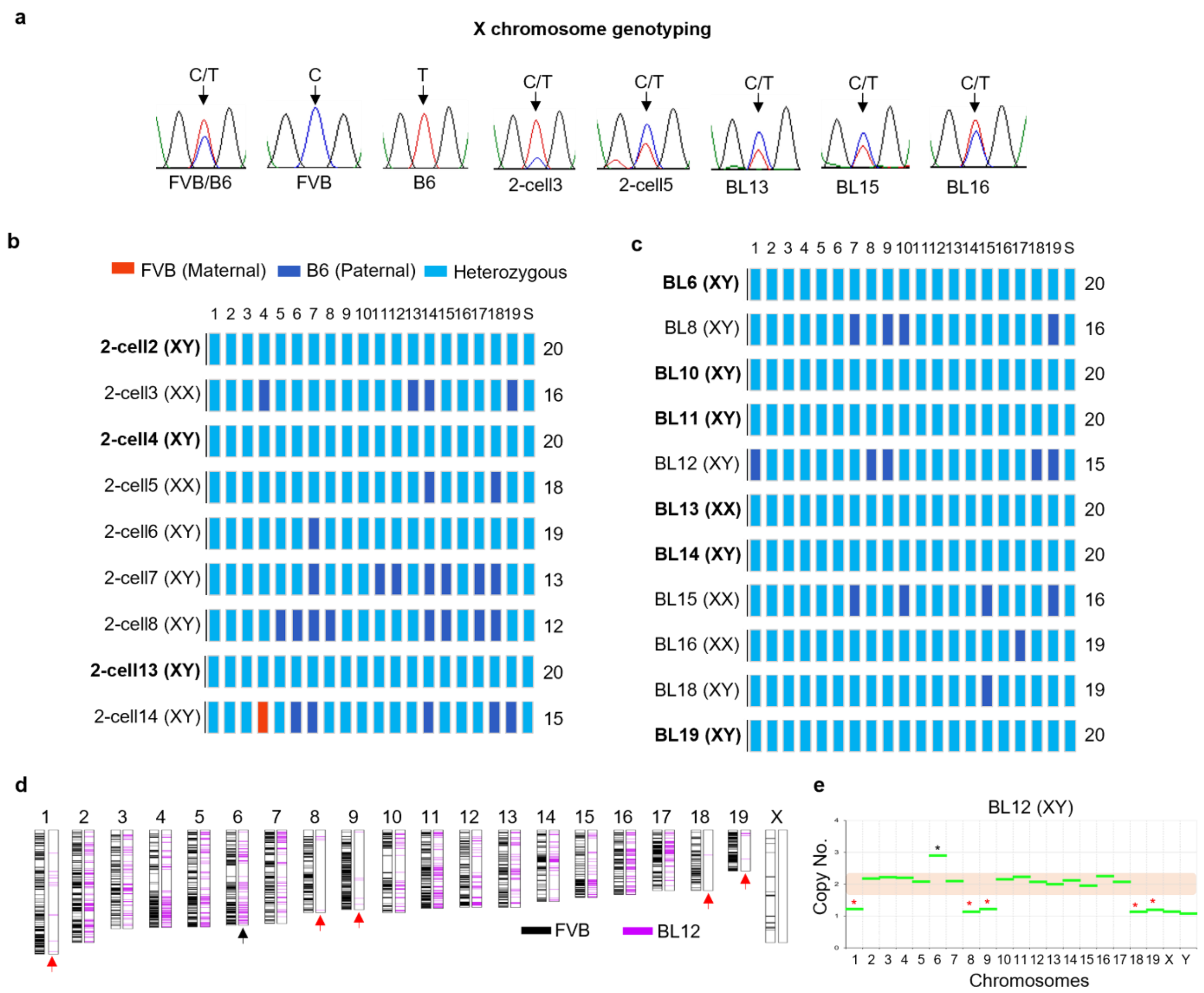

Supplementary Fig. 7. Contribution of the somatic origin in preimplantation embryos. a, X chromosome genotyping in female SH 2-cells and SH-blastocysts by Sanger sequencing. All-female embryos showed heterozygous peaks in chromatography. **b and c,** Chromosome genotypes in SH 2-cells and SH-blastocysts. Red and blue bars mean FVB (maternal) and B6 (paternal) genotype, respectively. Light blue bars indicated heterozygous status (FVB/B6). S above the last bars indicate sex chromosome. SH 2-cell 2, 4, and 13 and SH-blastocyst 6, 10, 11, 13, 14, and 19 (bold letters) showed heterozygosity in all 20 chromosomes. **d,** Chromosome map of the SH blastocysts 12. Blastocyst 12 showed the B6 genotype in chromosomes 1, 8, 9, 18, and 19 (red arrows). Chromosome 6 of blastocyst 6 (black arrow) displayed FVB genomes but was triploid

based on CNV analysis. **e,** CNV profile of the SH blastocysts 12 with exome data. Chromosomes 1, 8, 9, 18, and 19 were haploid (red asterisk), whereas chromosome 6 was triploid (black asterisk). Relative CNV was interpreted by comparison with the control *in vitro* fertilization-embryo, second polar body, and C57BL/6 mouse tissue as a control.

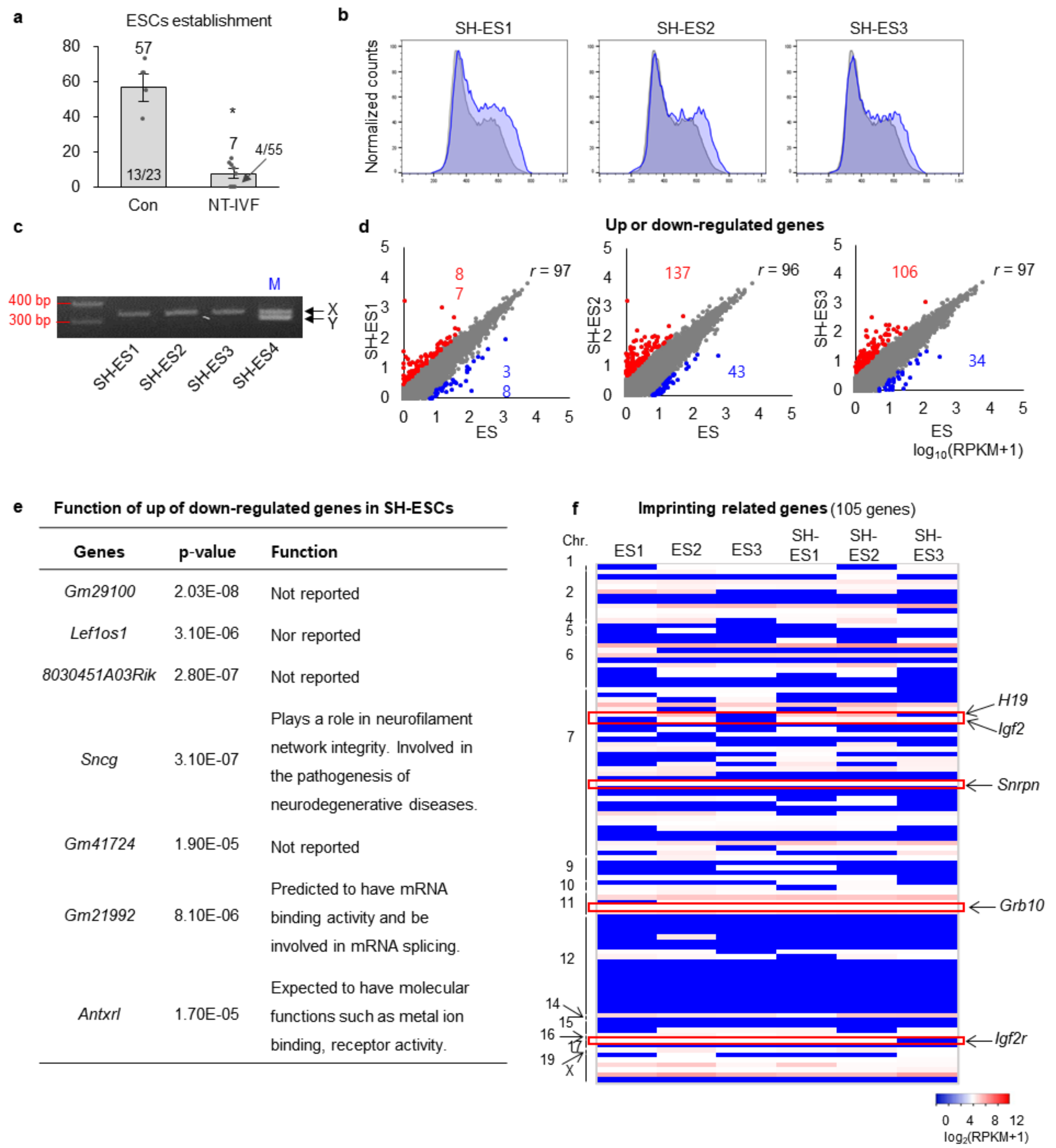

Genes	p-value	Function
Gm29100	2.03E-08	Not reported
Lef1os1	3.10E-06	Nor reported
8030451A03Rik	2.80E-07	Not reported
Sncg	3.10E-07	Plays a role in neurofilament network integrity. Involved in the pathogenesis of neurodegenerative diseases.
Gm41724	1.90E-05	Not reported
Gm21992	8.10E-06	Predicted to have mRNA binding activity and be involved in mRNA splicing.
Antxrl	1.70E-05	Expected to have molecular functions such as metal ion binding, receptor activity.

Supplementary Fig. 8. Transcriptional analysis of SH-ESCs. a, The efficiency of SH-ESCs derivation. The efficiency was 7%, which was significantly lower than that of ESC from IVF embryos (57%). n means the number of established ESCs/the number of plated blastocysts. 4 or 7 technical replications for control and NT-IVF group, respectively. mean ± s.e.m. ($P < 0.05$, by

Independent-group t-test). **b,** Diploid configuration of the SH-ESC lines by cell cycle analysis. The histogram refers to the cell cycle profile of the SH-ESC lines resulting in a 2n nuclear configuration. **c,** Sex identification. Only SH-ES4 was male. Letter M above the gel image means male. **d,** The number of up or down-regulated genes in each SH-ESC line compared to intact ESCs. **e,** Function of up or down-regulated genes in SH-ESCs. **f,** Heat map displaying imprinting related genes (105 genes) in intact ESCs and SH-ESCs. All genes showed no significant difference in SH-ESCs to intact ESCs ($p>0.05$).

a

***In vitro* development with various type of donor cell and sperm**

Lab	Experiment	Donor	Sperm	Oocytes N	Fertilized N (%)	2PN formation N (%)	2PN/PPB formation N (%)	2-cells N (%)	Morula N (%)	Blastocysts N (%)
E.K	Intact-IVF	-	B6	227	218 (96)	210 (96)	206 (94)	199 (97)	152 (74)	138 (67)
	NT-IVF	FVB MEF	B6	282	278 (99)	212 (76)	188 (68)	180 (96)	92 (49)	29 (15)
		FVB adult fib	B6	228	225 (99)	191 (85)	147 (65)	142 (97)	55 (37)	25 (17)
		B6/FVB MEF	B6	202	198 (98)	142 (72)	129 (65)	127 (98)	52 (40)	23 (18)
		Subtotal	-	712	701 (98)	545 (78)	464 (66)	449 (97)	199 (43)	77 (17)
G.D.P	Intact ICSI	-	BDF1	545	406 (74)	-	345 (85)	-	-	289 (84)
	NT-ICSI	BDF1 cumulus	BDF1	-	413	-	271 (66)	-	-	81 (30)
S.M	NT-IVF	FVB cumulus	B6	1449	-	-	1008 (70)*	970 (96)	230 (23)	111 (11)

b

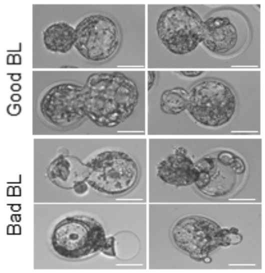

c

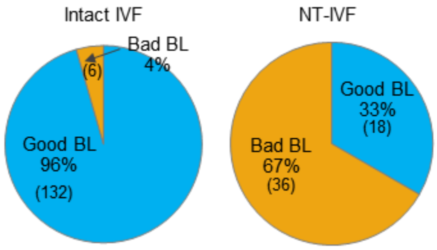

d

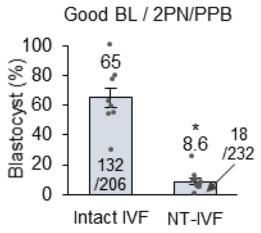

e **Results of aCGH in blastocysts**

Sample ID	Normal	Ploidy
CC-BL1	Yes	38XX
CC-BL2	No	38XX (+14p,+10q, +9q)
CC-BL3	No	38XY(+10p)
CC-BL4	Yes	38 XY
CC-BL5	No	37XX, mos+5, +8q, +9
CC-BL6	No	38XX, -14, +16, mos+17
CC-BL7	No	42XY, +2,+6,+8,+10,+13,-14,
CC-BL8	Yes	38 XX
CC-BL9	No	38X0, +8, -X

f

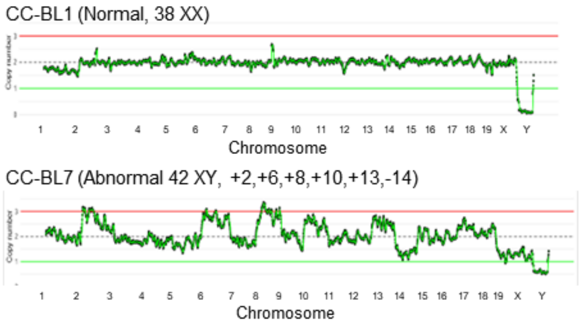

g

Somatic cells Type	Somatic cells Strain	Sperm Strain	Transferred SH-Blastocysts N	Total Recipient N	Pregnancy N	Offspring N
Fibroblasts	FVB	B6	95	9	0	0
	B6/FVB	B6	118	10	0	0
	B6/FVB	BDF1	121	35	3	3 (implanted)
Cumulus cells	BDF1	BDF1	81	27	1	3 (live)
ICSI control			35	5	4	23 (live)

h

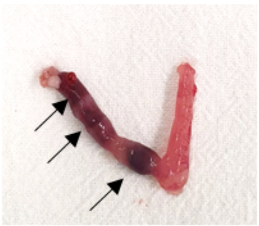

i

Pup #	Sexing	Birth weight (g)
1	Female	1.15
2	Female	1.23
3	Female	0.92
ICSI control		1.66 ± 0.17

j

SCNT Pup # (Gender)	No. of Offspring	Offspring Gender	Birth weight (g)	Weaned
1 (Female)	5	3F 2M	1.6 ± 0.3	5
2 (Female)	4	3F 1M	1.7 ± 0.1	4
3 (Female)	4	3F 1M	1.5 ± 0.2	4

Supplementary Fig. 9. Somatic haploid embryos are able to produce live offspring. a, Development of preimplantation embryos with various somatic donor cells and sperm in the independent laboratories. % of fertilization, calculated based on the number of oocytes; % of 2PN or 2PN/1PPB formation, calculated based on the number of fertilized oocytes; % of 2-cell, morula, and blastocysts, calculated based on the number of 2PN/1PPB embryos; *, calculated based on the number of oocytes; E.K, G.D.P, and S.M, Eunju Kang, Gianpiero D. Palermo, and Shoukhrat Mitalipov lab. **b,** Morphology of SH-blastocysts with good and poor quality. Good blastocysts were composed of many cells of ICM and trophectoderm, while poor blastocysts showed few ICM and trophectoderm. Scale bar: 40 μm. **c,** Ratio of blastocysts with good and poor quality in intact control and NT-IVF. **d,** Lower development into good blastocysts in NT-IVF. n indicates the number of good blastocysts/the number of 2PN/PPB embryos. 10 or 11 technical replications for intact IVF and NT-IVF groups, respectively. mean ± s.e.m. * $P < 0.05$, by Independent-group t-test. **e,** Ploidy analysis of SH-blastocysts using aCGH. Three blastocysts (CC-BL1, 4, and 8) were euploid among nine blastocysts. **f,** aCGH profile of euploid (CC-BL1) and aneuploid (CC-BL7) SH-blastocyst. **g,** Different pregnancy and offspring generations depending on the strain of the somatic cell and sperm. The hybrid combination of FVB/B6 and the BDF1 hybrid somatic cells with BDF1 hybrid sperm resulted in implantation or pup generation. **h,** Implantation of SH-embryos in the recipient uterus. Black arrows indicate implanted spot of SH-embryos on day seven after embryo transfer. **i,** Sexing and birth weight in SH-mice. Mean ± s.d. **j,** The information in the first generation of SH-mice. Mean ± s.d; F, fammale; M, male.

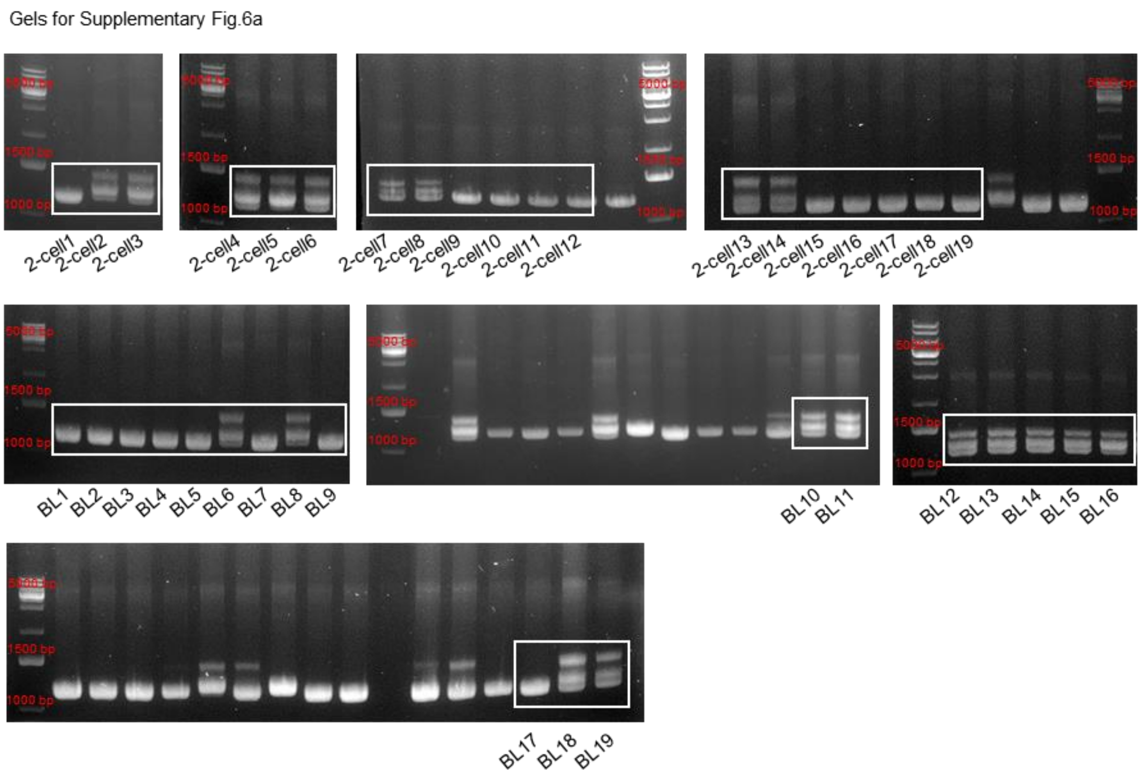

Supplementary Fig. 10. Uncropped gel images for supplementary Fig. 6a

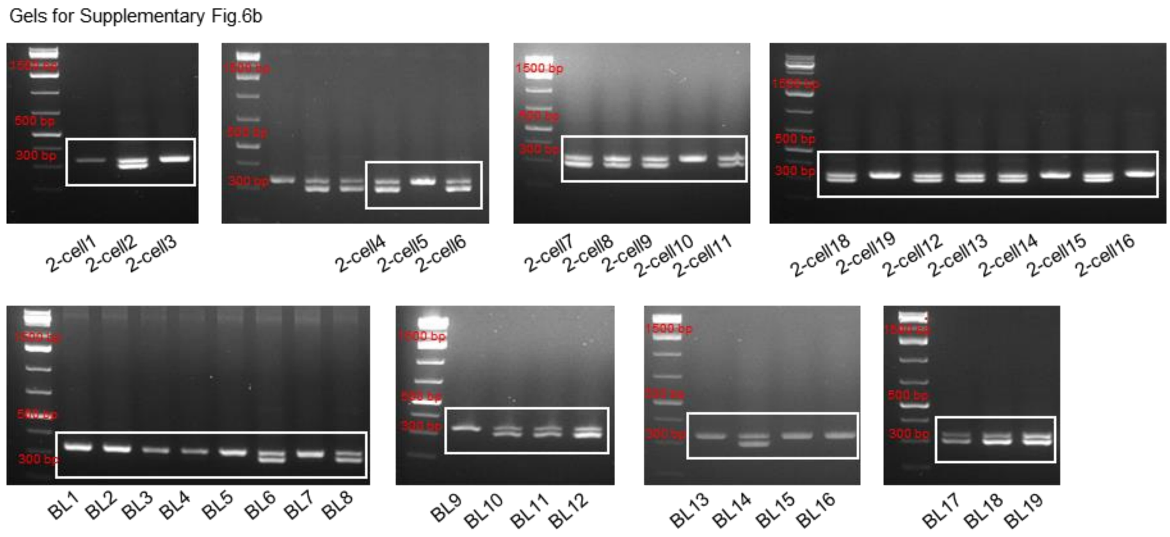

Supplementary Fig. 11. Uncropped gel images for supplementary Fig. 6b

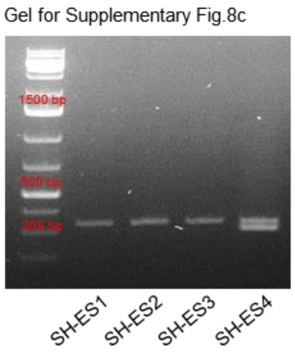

Supplementary Fig. 12. Uncropped gel image for supplementary Fig. 8c

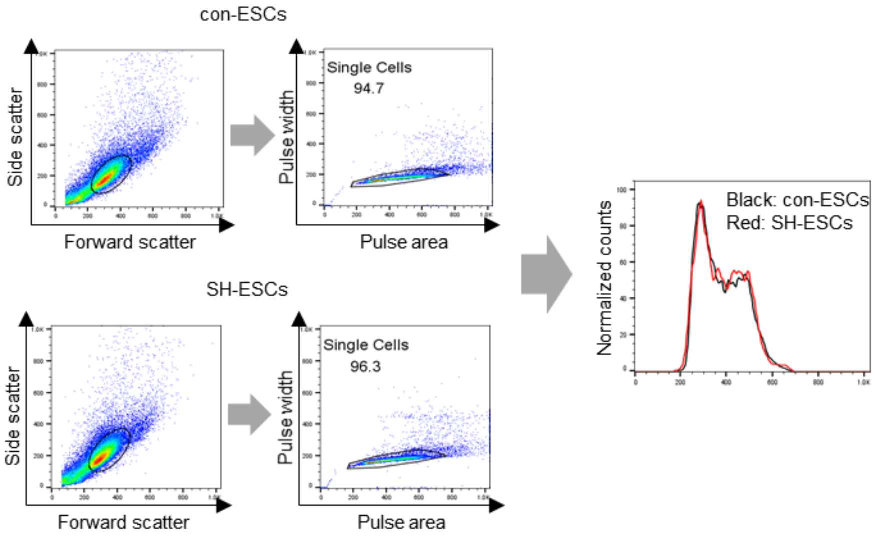

Supplementary Fig. 13. The gating strategy of flow cytometry. The gating was applied to the scatter plot. Debris and death cells were gated out and single cells were gated using pulse width versus pulse area plot. All samples were applied the same strategy. These plots are relative to Figure 6C as an example for the gating strategy.

Supplementary Table. 1. Development of preimplantation embryos with various chemical treatments.

Group	Resting time after SCNT				Treatment during IVF	Treatment during overnight	Oocytes N	Fertilized N (%)	2PN formation N (%)	2PN/1PPB formation N (%)	2-cells N (%)	Morula N (%)	Blastocysts N (%)
	Total	RA treatment	RA time	RS-1 treatment									
Intact-IVF							227	218 (96)	210 (96)	206 (94)	199 (97)	152 (76)	138 (91)
Regular SCNT							60	-	39 (65)	-	32 (82)	16 (50)	11 (69)
NT-IVF	0.5 h						117	116 (99)	36 (31)	0 (0)	-	-	-
	1 h						81	78 (96)	29 (37)	3 (4)	-	-	-
	1.5 h						91	88(97)	56 (64)	11 (13)	-	-	-
	2 h						95	87 (92)	66 (76)	16 (19)[a]	-	-	-
	3 h						87	86 (99)	39 (45)	8 (9)	-	-	-
	2 h						198	184 (93)	144 (78)	31 (17)	29 (94)	14 (48)	4 (29)
					Sc	Sc	111	109 (98)	79 (72)	24 (22)	23 (96)	10 (43)	4 (40)
					Sc+Fa	Sc+Fa	187	184 (98)	142 (77)	58 (32)[b]	54 (93)	26 (48)	10 (38)
					Sc+Fa	Sc+Fa	112	108 (96)	79 (73)	35 (32)	-	-	-
					Subtotal	-	312	305 (98)	230 (75)	103 (34)	-	-	-
		RA	0.5 h		Sc+Fa	Sc+Fa	92	89 (97)	63 (71)	46 (52)[c]	-	-	-
		RA	1 h		Sc+Fa	Sc+Fa	94	83 (88)	53 (64)	20 (24)	-	-	-
		RA	2 h		Sc+Fa	Sc+Fa	78	70 (90)	45 (64)	17 (24)	-	-	-
		RA	0.5 h		Sc+Fa	Sc+Fa	208	202 (97)	144 (71)	104 (51)[d]	95 (91)	40 (42)	20 (50)
				RS-1	Sc+Fa	Sc+Fa	155	153 (99)	117 (76)	62 (40)	57 (92)	23 (40)	6 (26)
		RA	0.5 h	RS-1	Sc+Fa	Sc+Fa	158	155 (98)	123 (79)	78 (50)	73 (94)	26 (36)	8 (31)
		RA	0.5 h		Sc+Fa+RS-1		124	118 (95)	102 (87)	82 (69)[e]	74 (90)	27 (36)	9 (33)
		RA	0.5 h		Sc+Fa+RS-1	Sc+Fa+RS-1	229	221 (97)	180 (81)	148 (67)[e]	135 (91)	54 (40)	27 (50)
		RA	0.5 h	RS-1	Sc+Fa+RS-1	Sc+Fa+RS-1	188	183 (97)	124 (68)	72 (39)	68 (94)	23 (34)	11 (48)

Percentages of fertilization were calculated based on the number of oocytes. Percentages of 2PN or 2PN/1PPB formation were calculated based on the number of fertilized oocytes for intact IVF and NT-IVF. Percentages of 2PN were calculated based on the number of oocytes for regular SCNT. Percentages of 2-cell, morula, and blastocysts were calculated based on the number of the previous embryo stage.

[a] The rate of 2PN/1PPB formation was significantly higher in 2 hrs among different resting times after SCNT ($P < 0.05$, by Independent-group t-test).

[b] 2PN/1PPB rate was significantly higher in a fasudil-treated group (Sc+Fa) than the non-treated group (Sc) ($P < 0.05$, by Independent-group t-test).

[c] Treatment with RA for 30 min displayed the highest 2PN/1PPB rate compared with 1 h and 2 h treatment ($P < 0.05$, by Independent-group t-test).

[d] The 2PN/1PPB rate in the RA-treated group (Sc+Fa and RA) was significantly higher than that in the untreated group (Only Sc+Fa), ($P < 0.05$, by Independent-group t-test).

[e] RS-1 treatment was applied during the resting time after SCNT, during IVF, and overnight. The 2PN/1PPB rate was significantly higher during IVF compared to treatment during the resting time or both resting and IVF ($P < 0.05$, by ANOVA with Tukey analysis). Further, RS-1 treatment during IVF increased the 2PN/1PPB rate significantly than treatment only RA during the resting time ($P < 0.05$, by Independent-group t-test).

Supplementary Table 2. The efficiency of embryo development with various chemical treatments.

Group	Resting time after SCNT		Treatment during IVF	Treatment during overnight	Oocytes N	Fertilization %	2PN/1PPB formation %	2-cells %	Morula %	Blastocysts %
	RA treatment	RS-1 treatment								
Intact-IVF					227	96	90	88	67	61
					198	93	16	15	7	2
			Sc	Sc	111	98	22	21	9	4
			Sc+Fa	Sc+Fa	187	98	31	29	14	5
	RA		Sc+Fa	Sc+Fa	208	97	49	45	19	9
NT-IVF		RS-1	Sc+Fa	Sc+Fa	155	99	40	36	15	4
	RA	RS-1	Sc+Fa	Sc+Fa	158	98	49	46	17	5
	RA		Sc+Fa+RS-1		124	95	66^{*}	59	21	7
	RA		Sc+Fa+RS-1	Sc+Fa+RS-1	229	97	65^{*}	59	24	$12^{\#}$
	RA	RS-1	Sc+Fa+RS-1	Sc+Fa+RS-1	188	97	38	36	12	6

The efficiency was calculated by a percentage of a percentage. * or $^{\#}$ indicate significantly higher 2PN/1PPB or blastocyst rate compared to non-treatment groups ($P < 0.05$, by Independent-group t-test).
