# Supplementary material for: Haploidy in somatic cells is induced by mature oocytes in mice
Source: Commun Biol. 2022 Jan 25;5:95. doi: 10.1038/s42003-022-03040-5 (PMC8789866; doi:10.1038/s42003-022-03040-5)
Supplement: Supplementary file 3 — Description of Additional Supplementary Files [file 42003_2022_3040_MOESM3_ESM.pdf]

Description of Additional Supplementary Files

File name: Supplementary Movie 1
Description: The process of NT-IVF and the development of SH embryos.

MII oocytes were secured with a holding pipette and the zona pellucida was drilled with a laser. An enucleation pipette was inserted into the cytoplasm, and a small amount of cytoplasm containing the spindle was removed from the oocyte. Next, an HVJ-E extract-treated donor cell was aspirated into a micropipette and transferred into the enucleated oocyte. The time-lapse video was taken under polarized light microscopy using Oosight™ software displaying the pseudo-meiotic-like spindle development following the nuclear transfer of a cumulus cell into an enucleated oblast. The full development of preimplantation SH-embryo generated by NT-ICSI was observed using a time-lapse imaging system.

File name: Supplementary Data 1
Description: The source data for generating the graphs and charts are shown in the main figures.

File name: Supplementary Data 2
Description: Nuclear variants of FVB/N, C57BL/6, and DBA/2 mice, and SH-embryos by whole-exome sequencing.

The exome data were aligned to the C57BL/6 genome sequence reference (GCF_000000055.19, GRCm38.p6). 0/1 and 1/1 indicate heterozygosity and homozygosity of altered SNPs, respectively. The data could include errors due to the sequencing process.

File name: Supplementary Data 3
Description: Genotyping of intact IVF embryos and SH-embryos by Miseq.

Red letters in sheet 1 could be the extrusion of two-pair of chromatids into the unseparated chromosomes into PPBs in NT-IVF embryos. The blue letters in sheet 1 could be errors of whole genome amplification. Asterisk indicates the genotype by Sanger sequencing in sheet 1. N/A could be no extraction of unseparated chromosomes or errors of whole genome amplification in sheet 1.
