# Supplementary material for: Haploidy in somatic cells is induced by mature oocytes in mice
Source: Commun Biol. 2022 Jan 25;5:95. doi: 10.1038/s42003-022-03040-5 (PMC8789866; doi:10.1038/s42003-022-03040-5)
Supplement: Supplementary file 8 — Reporting Summary [file 42003_2022_3040_MOESM8_ESM.pdf]

nature portfolio

Corresponding author(s): Eunju Kang
Shoukhrat Mitalipov
Gianpiero D. Palermo

Last updated by author(s): Jan 3, 2022

Reporting Summary

Nature Portfolio wishes to improve the reproducibility of the work that we publish. This form provides structure for consistency and transparency in reporting. For further information on Nature Portfolio policies, see our Editorial Policies and the Editorial Policy Checklist.

Statistics

For all statistical analyses, confirm that the following items are present in the figure legend, table legend, main text, or Methods section.

n/a	Confirmed	
☐	☒	The exact sample size (*n*) for each experimental group/condition, given as a discrete number and unit of measurement
☐	☒	A statement on whether measurements were taken from distinct samples or whether the same sample was measured repeatedly
☐	☒	The statistical test(s) used AND whether they are one- or two-sided<br>*Only common tests should be described solely by name; describe more complex techniques in the Methods section.*
☐	☒	A description of all covariates tested
☒	☐	A description of any assumptions or corrections, such as tests of normality and adjustment for multiple comparisons
☐	☒	A full description of the statistical parameters including central tendency (e.g. means) or other basic estimates (e.g. regression coefficient) AND variation (e.g. standard deviation) or associated estimates of uncertainty (e.g. confidence intervals)
☒	☐	For null hypothesis testing, the test statistic (e.g. *F*, *t*, *r*) with confidence intervals, effect sizes, degrees of freedom and *P* value noted<br>*Give P values as exact values whenever suitable.*
☒	☐	For Bayesian analysis, information on the choice of priors and Markov chain Monte Carlo settings
☒	☐	For hierarchical and complex designs, identification of the appropriate level for tests and full reporting of outcomes
☒	☐	Estimates of effect sizes (e.g. Cohen's *d*, Pearson's *r*), indicating how they were calculated

Our web collection on statistics for biologists contains articles on many of the points above.

Software and code

Policy information about availability of computer code

Data collection	No software was used.
Data analysis	No software was used.

For manuscripts utilizing custom algorithms or software that are central to the research but not yet described in published literature, software must be made available to editors and reviewers. We strongly encourage code deposition in a community repository (e.g. GitHub). See the Nature Portfolio guidelines for submitting code & software for further information.

Data

Policy information about availability of data

All manuscripts must include a data availability statement. This statement should provide the following information, where applicable:
- Accession codes, unique identifiers, or web links for publicly available datasets
- A description of any restrictions on data availability
- For clinical datasets or third party data, please ensure that the statement adheres to our policy

All data supporting the findings of the current study are provided in the paper and Supplementary Information. All additional information will be made available upon request to the authors.

Field-specific reporting

Please select the one below that is the best fit for your research. If you are not sure, read the appropriate sections before making your selection.

☒ Life sciences ☐ Behavioural & social sciences ☐ Ecological, evolutionary & environmental sciences

For a reference copy of the document with all sections, see nature.com/documents/nr-reporting-summary-flat.pdf

Life sciences study design

All studies must disclose on these points even when the disclosure is negative.

Sample size	The number of oocytes used for somatic cell nuclear transfer are a minimum of 60 to a maximum of 1449 in each group.
Data exclusions	No data were excluded from the analysis.
Replication	All attempts at replication were successful.
Randomization	Multiple cell lines were isolated from mice and randomly selected and used for the experiments.
Blinding	Investigators were blinded to group allocation during data analysis.

Reporting for specific materials, systems and methods

We require information from authors about some types of materials, experimental systems and methods used in many studies. Here, indicate whether each material, system or method listed is relevant to your study. If you are not sure if a list item applies to your research, read the appropriate section before selecting a response.

Materials & experimental systems

n/a	Involved in the study	
☐	☒	Antibodies
☐	☒	Eukaryotic cell lines
☒	☐	Palaeontology and archaeology
☐	☒	Animals and other organisms
☒	☐	Human research participants
☒	☐	Clinical data
☒	☐	Dual use research of concern

Methods

n/a	Involved in the study	
☒	☐	ChIP-seq
☐	☒	Flow cytometry
☒	☐	MRI-based neuroimaging

Antibodies

Antibodies used	Anti-Centromere antibody (Antibodies Incorporated, cat# 15–234)<br>Goat anti-Human IgG (H+L) Cross-Adsorbed Secondary Antibody, Alexa Fluor 568 (Thermo Fisher Scientific, cat# A-21090)<br>α-Tubulin (11H10) Rabbit mAb (Alexa Fluor® 488 Conjugate) (Cell Signaling Technology, cat# 5063)
Validation	The control samples expressing the antibodies were stained to confirm expression in this study.

Eukaryotic cell lines

Policy information about cell lines

Cell line source(s)	Fibroblasts from FVB mice and hybrid mice (FVB/B6 and B6/FVB mice) and somatic haploidization embryonic stem cell lines
Authentication	Genome sequencing was performed to confirm the genotype of cell lines.
Mycoplasma contamination	All cell lines were tested negative for mycoplasma contamination.
Commonly misidentified lines<br>(See ICLAC register)	None for this study.

Animals and other organisms

Policy information about studies involving animals; ARRIVE guidelines recommended for reporting animal research

Laboratory animals	Mouse B6D2F1 male 8-9 weeks old<br>Mouse B6D2F1 female 8-9 weeks old<br>Mouse C57BL/6 male 8-9 weeks old<br>Mouse C57BL/6 female 8-9 weeks old<br>Mouse FVB/N male 8-9 weeks old<br>Mouse FVB/N female 8-9 weeks old<br>Mouse female ICR 10-12 weeks old<br>Mouse NOD-Prkdcem1Baek Il2rgem1Baek (NOD-SCID Gamma, NSG) 7 weeks old
Wild animals	The study did not involve wild animals.
Field-collected samples	The mice were housed under a 12-hour shift of light/dark cycle under pathogen-free conditions with free access to water and food. Mice were euthanized to collect experimental materials, such as oocyte and sperm.
Ethics oversight	All animal maintenance and experimental procedures were performed following the guidelines of the Asan Medical Center, Oregon Health & Science University, and Weill Cornell Medicine. The animal protocols were reviewed and approved by the Asan Medical Center Institute, Small Lab Animal Unit at Oregon Health & Science University, and Weill Cornell Medicine Institutional Animal Care and Use Committee (IACUC) before any experiments were performed.

Note that full information on the approval of the study protocol must also be provided in the manuscript.

Flow Cytometry

Plots

Confirm that:

☒ The axis labels state the marker and fluorochrome used (e.g. CD4-FITC).

☒ The axis scales are clearly visible. Include numbers along axes only for bottom left plot of group (a 'group' is an analysis of identical markers).

☒ All plots are contour plots with outliers or pseudocolor plots.

☒ A numerical value for number of cells or percentage (with statistics) is provided.

Methodology

Sample preparation	somatic haploidization embryonic stem cell lines generated in this study
Instrument	FACSCalibur, BD Biosciences
Software	FlowJo v10.5.3
Cell population abundance	10,000 single cell events were adjusted in the pulse width versus pulse area plot. The purity of single cell events was greater than 90 % excluding debris and death cell populations. The purity was determined by the gating tool in FlowJo software.
Gating strategy	Single cell populations were gated using pulse width versus pulse area plot. The gate was applied to the scatter plot and debris and death cells were gated out. The plot was applied to the PI histogram plot. All samples were applied the same gating strategy.

☒ Tick this box to confirm that a figure exemplifying the gating strategy is provided in the Supplementary Information.
